# Supplementary material for: Ant colonies maintain social homeostasis in the face of decreased density
Source: eLife. 2019 May 2;8:e38473. doi: 10.7554/eLife.38473 (PMC6497443; doi:10.7554/eLife.38473)
Supplement: Table 1—source code 1. [file elife-38473-table1-code1.pdf]

```
cum.int=read.csv("cumulative_interactions_final.csv")
str(cum.int)
cum.int$colony=as.factor(cum.int$colony)

fit.pois=glm(N.start.of.interactions.at.time.x~colony*treatment,data= cum.int,family="pois
son")
summary(fit.pois)

library(multcomp)
int.mat=model.matrix(~0+colony*treatment,data=cum.int)
tmp=expand.grid(colony=unique(cum.int$colony),treatment=unique(cum.int$treatment))
tmp
X=model.matrix(~colony*treatment,data=tmp)
X
gg=glht(fit.pois,linfct=X)
gg
## checking that this is doing the right thing:
predict(fit.pois,newdata=tmp)

## making contrast matrix
K=matrix(c(1,0,0,-1,0,0,
          0,1,0,0,-1,0,
         0,0,1,0,0,-1),nrow=3,ncol=6,byrow=TRUE)
K
colnames(K)=c("1h","2h","3h","1l","2l","3l")
rownames(K)=c("C1:h-l","C2:h-l","C3:h-l")
summary(glht(fit.pois,linfct=K%*%X))
```
