# Supplementary material for: Ant colonies maintain social homeostasis in the face of decreased density
Source: eLife. 2019 May 2;8:e38473. doi: 10.7554/eLife.38473 (PMC6497443; doi:10.7554/eLife.38473)
Supplement: Table 1—source data 2. [file elife-38473-table1-data2.docx]

Source data 2. Simultaneous Tests for General Linear Hypotheses

Fit: glm(formula = N.start.of.interactions.at.time.x ~ colony * treatment,

    family = "poisson", data = [cum.int](http://cum.int/))

|  | Estimate | Standard Error | z-value | Pr(>\|z\|) |
| --- | --- | --- | --- | --- |
| Colony 1:high-low | -0.20030 | 0.06055 | -3.308 | 0.00282 |
| Colony 2:high-low | -0.54704 | 0.07024 | -7.788 | 2.07e-14 |
| Colony 3:high-low | 0.09487 | 0.05581 | 1.700 | 0.24440 |

Adjusted p-values reported -- single-step method
